# Ameliorative Potential of L-Alanyl L-Glutamine Dipeptide in Colon Cancer Patients Receiving Modified FOLFOX-6 Regarding the Incidence of Diarrhea, the Treatment Response, and Patients’ Survival: A Randomized Controlled Trial

**DOI:** 10.3390/medicina58030394

**Published:** 2022-03-07

**Authors:** Nesreen M. Sabry, Tamer M. Naguib, Ahmed M. Kabel, El-Sayed Khafagy, Hany H. Arab, Walid A. Almorsy

**Affiliations:** 1 Clinical Oncology Department, Faculty of Medicine, Tanta University, Tanta 31527, Egypt; nesreensabry1eg@yahoo.com (N.M.S.); walidaa1@hotmail.com (W.A.A.); 2 Anesthesia and ICU Department, Faculty of Medicine, Tanta University, Tanta 31527, Egypt; tnaguib1eg@yahoo.com; 3 Department of Pharmacology, Faculty of Medicine, Tanta University, Tanta 31527, Egypt; 4 Department of Pharmaceutics, College of Pharmacy, Prince Sattam Bin Abdulaziz University, Al-Kharj 11942, Saudi Arabia; e.khafagy@psau.edu.sa; 5 Department of Pharmaceutics and Industrial Pharmacy, Faculty of Pharmacy, Suez Canal University, Ismailia 41522, Egypt; 6 Department of Pharmacology and Toxicology, College of Pharmacy, Taif University, P.O. Box 11099, Taif 21944, Saudi Arabia; h.arab@tu.edu.sa

**Keywords:** colon cancer, L-alanyl L-glutamine dipeptide, FOLFOX, diarrhea, patients’ survival

## Abstract

*Background and Objectives*: Diarrhea induced by chemotherapy may represent a life-threatening adverse effect in cancer patients receiving chemotherapy. FOLFOX, an effective treatment for colon cancer, has been associated with diarrhea with high severity, particularly with higher doses. Management of diarrhea is crucial to increase the survival of cancer patients and to improve the quality of life. Glutamine is an abundant protein peptide found in blood and has a crucial role in boosting immunity, increasing protein anabolism, and decreasing the inflammatory effects of chemotherapy on the mucosal membranes, including diarrhea. This study aimed to provide evidence that parenteral L-alanyl L-glutamine dipeptide may have a positive influence on the incidence of diarrhea, treatment response, and the overall survival in colon cancer patients treated with modified FOLFOX-6 (mFOLFOX-6). *Materials and Methods*: Forty-four stage II and III colon cancer patients were included in this study where they were treated with the standard colon cancer chemotherapy mFOLFOX-6 and were randomly allocated into glutamine group and placebo group, each of 22 patients. *Results*: L-alanyl L-glutamine dipeptide was found to be significantly effective in decreasing the frequency and severity of diarrhea when compared to the placebo group, particularly after four and six cycles of mFOLFOX-6. There was no significant difference between the studied groups regarding to the overall survival. *Conclusion*: L-alanyl L-glutamine dipeptide can be considered as an add-on with chemotherapy to improve the quality of life and the overall survival of colon cancer patients.

## 1. Introduction

L-alanyl L-glutamine dipeptide (DIP) is a water-soluble compound that is used for nutritional purposes where it acts locally in the gastrointestinal (GI) tract to keep the integrity of the mucosa and preserve the intestinal functions [1]. Consequently, the use of DIP may help in reducing the incidence of bacterial infections and inflammation of the intestine and preventing related symptoms, such as diarrhea [2]. DIP enteral administration was found to increase the mucosal and plasma levels of reduced glutathione (GSH). It may act via stimulation of synthesis of the mucosal proteins and may recover the protein balance of the intestine during stress conditions, such as cancer [3]. The effect of glutamine on cancer cells had been extensively studied. Glutamine represents a crucial metabolic substrate that is proven to be dysregulated in cancer [4]. Multiple studies had demonstrated the effect of glutamine as a protective agent by enhancing the immunity and improving the overall survival in cancer patients [5,6]. Jiang et al. [7] revealed that L-glutamine may enhance redox homeostasis through adaptation to anchorage independence in lung cancer. Another study that was carried out by Anderson and Lalla [8] revealed the positive effect of glutamine in preventing chemotherapy and radiation-induced mucositis.

In Egypt, colorectal malignancies represent about 6.5% of all malignant tumors and were reported among the most prevalent tumors [9]. The crude incidence rate in males was 3.1 for colon carcinoma and 1 for rectal carcinoma. In females, the crude incidence rate was 2.3 for colon carcinoma and 0.8 for rectal carcinoma [10].

Chemotherapy-associated diarrhea is common among cancer patients, particularly those who are treated with fluoropyrimidines e.g., fluorouracil [FU], as well as capecitabine and irinotecan, and is usually dose dependent [11]. FOLFOX (5-fluorouracil (5FU)/leucovorin with oxaliplatin) is a chemotherapy regimen used for treatment of advanced colon cancer [12] and is usually associated with diarrhea [13]. This may be due to direct cytotoxic effects on the mucosal lining of the gastrointestinal tract together with alteration of the intestinal microflora [11]. A study by Bano and colleagues revealed a 42% incidence of grade 2 stomatitis in patients treated with FOLFOX (oxaliplatin 100 mg/m^2^). In addition, administration of oxaliplatin (130 mg/m^2^) was associated with severe GI symptoms. Twenty-five percent of patients had grade 3 diarrhea while grade 4 diarrhea was detected in 4% of patients [14]. The Canadian Working Group on Chemotherapy-Induced Diarrhea reported that high doses of FOLFOX were associated with 66% incidence of either grade 3 or 4 diarrhea compared to other regimens used for treatment of colon cancer [15].

The present study assessed the positive effect of glutamine on prevention of diarrhea and improvement of the overall survival and treatment response in patients with colon cancer who received mFOLFOX-6.

## 2. Materials and Methods

### 2.1. Eligible Patients

This randomized controlled trial was registered on the ISRCTN registry with trial ID ISRCTN13489936 and was conducted in Clinical Oncology Department Tanta University, Egypt, after approval of the Research Ethics Committee of Faculty of Medicine, Tanta University (Approval code 34918/3; Date of approval 8 March 2019). The written informed consents were obtained from all the participants before being included in this study. The experimental protocol was carried out according to Helsinki declaration. A total of 44 colon cancer patients were included and randomly allocated into two equal groups; glutamine group and placebo group between April 2019 to April 2021.

### 2.2. Inclusion Criteria

Patients of both genders, aged ≥18 years with histologically confirmed colon adenocarcinoma; stage II, and III according to American Joint Committee on Cancer and the Union for International Cancer Control (AJCC-UICC); 7th Edition [16] were enrolled. ECOG performance state, adequate hematological (evidenced by white blood cell count ≥ 4000/μL and platelet count ≥ 100,000/μL), renal (creatinine < 1.5 mg/dL) and hepatic functions (serum total bilirubin < 1.5 mg/dL).

### 2.3. Exclusion Criteria

Patients with stage IV, second primary, or any other comorbidity were excluded from this study.

### 2.4. Treatment Plan (Chemotherapy)

All patients were treated with the standard mFOLFOX-6 consisting of 2 h intravenous (IV) infusion of oxaliplatin (85 mg/m^2^) on day 1, and 2 h IV drip infusion of calcium folinate (400 mg/m^2^) on day 1, followed by IV injection of 5-FU (400 mg/m^2^) and continuous infusion of 5-FU (1200 mg/m^2^) on days 1–2 (Total 2400 mg/m^2^ over 46–48 h). The intravenous infusion was continued every 2 weeks. Patients were randomized to receive glutamine dipeptide (Dipeptiven) *n* = 22; glutamine dipeptide group) or not receiving glutamine dipeptide (*n* = 22; control group). In the glutamine dipeptide group, (N(2)-L-Alanyl-L-Glutamine Dipeptide, (Dipeptiven), by Fresenius Laboratories, Bad Homburg, Germany) was given IV in a dose of 20 gm/100 mL on the day 1–2 regimen every 2 weeks.

### 2.5. Follow Up

The included patients enrolled in both groups were evaluated at the baseline (prior to chemotherapy) and after two, four and six cycles of treatment. Treatment response to chemotherapy was assessed every two cycles according to the Response Evaluation Criteria in Solid Tumors (RECIST) [17]. Treatment-related toxicities were estimated according to standard World Health Organization (WHO) criteria [18]. Diarrhea was graded according to the National cancer institute [19]. In case of diarrhea grades I and II, only supportive therapy was considered. Grade III diarrhea was managed with supportive therapy, IV fluids and hospitalization. Chemotherapy was postponed till complete recovery and the dose of chemotherapy was reduced. Regarding patients with grade IV diarrhea, they were admitted in the ICU and given IV fluids, supportive care, monitoring of electrolytes and chemotherapy was stopped until complete recovery with dose reduction in case of reinfusion.

### 2.6. Statistical Analysis

Statistical analysis was done by SPSS version 26 (IBM Inc., Chicago, IL, USA). Quantitative variables were presented as mean ± standard deviation (SD). Comparison between the two groups was carried out utilizing unpaired Student’s *t*-tests. Qualitative variables were presented as frequency and percentage (%) and were analyzed utilizing the chi-square test when appropriate. Kaplan–Meier curves and hazard ratios were used to compare the survival rate between both groups. A two-tailed *p*-value ≤ 0.05 was considered statistically significant.

## 3. Results

**Figure 1:**
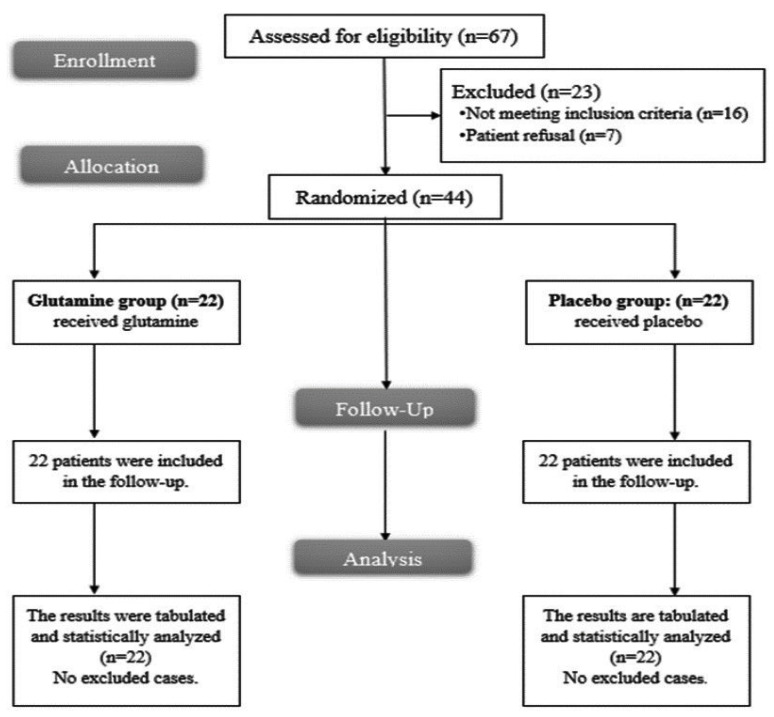
The randomized trial flow diagram, including enrollment, intervention allocation, follow-up, and analysis.

In the present study, 67 patients were assessed for eligibility. Sixteen patients did not meet the inclusion criteria and seven patients refused to participate in the study. Forty-four patients were randomly allocated into two equal groups: the glutamine group and placebo group. The patients were followed up and analyzed statistically as presented in Figure 1.

According to Table 1, patients in the glutamine group were significantly younger compared to the placebo group (*p*-value < 0.001). However, gender, physical status, carcino-embryonic antigen (CEA), and the stage of cancer were insignificantly different between both groups. All the studied patients had adenocarcinoma of the colon, underwent surgery and were subjected to mFOLFOX-6 chemotherapy.

Treatment response evaluation in both groups didn’t reveal any significant differences between both groups as presented in Table 2.

After two cycles of treatment with mFOLFOX-6, diarrhea was insignificantly different between both groups. After four and six cycles, diarrhea was significantly lower in glutamine group compared to placebo group (*p*-value < 0.001) as presented in Table 3.

**Figure 2:**
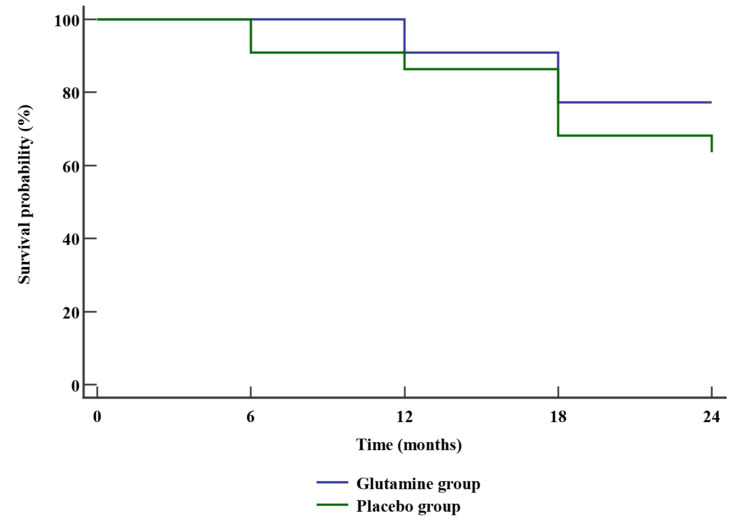
Kaplan–Meier curve for overall survival.

The overall survival in glutamine group was insignificantly different between glutamine and placebo groups. The hazard ratio of mortality in glutamine group was 0.56 times (95% CI: 0.18–1.76) lower than placebo group. The mortality rate was insignificantly different between glutamine group and placebo group (22.7% vs. 36.4% respectively, *p*-value = 0.509) as presented in Table 4 and Figure 2.

## 4. Discussion

Diarrhea associated with cancer chemotherapy may be severe enough to cause serious negative consequences such as malnutrition, dehydration, cardiovascular events, and even death. Additionally, diarrhea can cause chemotherapeutic dosing delays or reductions which may have an impact on the patient’s survival [11]. Since 2001, FOLFOX was presented as a new and the most effective treatment for malignancies of the colon and rectum [20]. A number of studies assessed the efficacy as well as the potential tolerability associated with different concentrations of FOLFOX such as FOLFOX-4, modified FOLFOX-4, FOLFOX-6, and modified FOLFOX-6 in colon cancer patients [21].

DIP has been widely introduced to cancer patients as a nutritional supplement to upsurge proliferation and survival under metabolic stress [6]. Additionally, glutamine was proven to be effective when used in combination with the immunosuppressive agents after bone marrow transplantation to compensate for the body protein waste and mucosal injury as well as, after high-dose chemotherapy [22]. As well, glutamine anabolic effect was found to fight against weight loss or sarcopenia to improve survival, increase lymphocytes, and prevent infection of the mucosa [23]. This may explain our study findings regarding the overall survival in patients who were administered glutamine. The mortality rate was insignificantly different between glutamine group and placebo group (22.7% vs. 36.4% respectively, *p*-value = 0.322).

On the contrary, glutamine, as the most abundant amino acid in plasma [6], is largely utilized by cancer cells for energy production and as a source of nitrogen for nucleic acid and amino acids synthesis as well [24]. This process which is known as glutaminolysis was investigated in clinical trials to understand cancer proliferation and progression [25] and to delineate its effect on cancer survival [26,27].

The incidence of diarrhea induced by chemotherapy is well established, particularly in patients on 5-fluorouracil. The rate of all grades of diarrhea during chemotherapy has been detected as high as 82% where about one third of the patients had diarrhea of the third or fourth grade [15]. In a phase II trial, a dose of intravenous oxaliplatin 50 mg/m^2^ (1 h infusion), Leucovorin 100 mg/m^2^ (1 h infusion) and 5-FU 2100 mg/m^2^ (24 h infusion) until disease progression was given for a total of eight cycles. They detected grade III diarrhea in about 66% of patients [28]. This comes in the same line with the results of the current study where grade II and grade III diarrhea were significantly detected more frequently in patients on placebo compared to those on glutamine supplementation after two and four cycles of chemotherapy. In addition, grade IV diarrhea was detected in patients on placebo and completely disappeared in glutamine group after six cycles of chemotherapy, as well as grade III. This was in agreement with Widjaja et al. [23] and Altman et al. [24] who reported that glutamine decreases the severity of diarrhea and mucositis associated with chemotherapy particularly with high doses via elevation of the lymphocytic count, reduction of gut permeability, changing the inflammatory pathways such as nuclear factor kappa B (NF-κB) and STAT signaling, augmentation of the defense mechanisms against apoptosis and oxidative stress, and preservation of tight-junction proteins [29,30].

Comparison of the treatment response in both groups didn’t reveal any significant differences. This may be explained by the controversial modulatory effect of glutamine on cancer cells since glutamine showed heterogenous metabolism in animals and tissue cultures [31]. Suzanne Klimberg and McClellan [32] recommended more trials to investigate the safety and efficacy of glutamine in cancer patients. Further, enhanced healing evidenced by the use of glutamine was reported to improve the quality of life and enable appropriate cancer treatment with lower incidence of associated adverse effects [23].

## 5. Conclusions

This study recommends the utilization of L-alanyl L-glutamine dipeptide as a supplementary treatment in colon cancer patients to decrease the severity of diarrhea and to improve the overall survival of these patients.

## Figures and Tables

**Table 1:** Patient characteristics of the studied groups.

		Glutamine Group (*n* = 22)	Placebo Group (*n* = 22)	*p*-Value
Age (years)	Mean ± SD	45.41 ± 5.72	53.09 ± 7.19	<0.001 *
	Range	35–61	44–66	
Gender	Male	7 (31.82%)	10 (45.45%)	0.390
	Female	15 (68.18%)	12 (54.55%)	
ECOG performance status	0	8 (36.36%)	4 (18.18%)	0.189
	1	9 (40.91%)	15 (68.18%)	
	2	5 (22.73%)	3 (13.64%)	
Site	Colon	22 (100%)	22 (100%)	-
Pathology	Adenocarcinoma	22 (100%)	22 (100%)	-
CEA	Mean ± SD	3.54 ± 1.82	4.18 ± 2.06	0.284
	Range	1–9	1–9	
Surgery	Yes	22 (100%)	22 (100%)	-
Stage	Stage II	5 (22.73%)	8 (36.36%)	0.322
	Stage III	17 (77.27%)	14 (36.64%)	
Treatment	Chemotherapy	22 (100%)	22 (100%)	-
Chemotherapy	mFOLFOX-6	22 (100%)	22 (100%)	-

CEA: carcinoembryonic antigen, *: significant as *p*-value ≤ 0.05.

**Table 2:** Response to treatment in the studied groups.

	Glutamine Group (*n* = 22)	Placebo Group (*n* = 22)	*p*-Value
Partial response	7 (31.82%)	6 (27.27%)	0.892
Complete response	15 (68.18%)	16 (72.73%)	

Data are presented as frequency (%).

**Table 3:** The incidence of diarrhea in the studied groups.

	Glutamine Group (*n* = 22)	Placebo Group (*n* = 22)	*p*-Value
After Two Cycles			
No diarrhea	12 (54.55%)	8 (36.36%)	0.066
Grade 1	8 (36.36%)	5 (22.73%)	
Grade 2	2 (9.09%)	7 (31.82%)	
Grade 3	0 (0%)	2 (9.09%)	
After Four Cycles			
No diarrhea	8 (36.36%)	1 (4.55%)	< 0.001 *
Grade 1	11 (50%)	2 (9.09%)	
Grade 2	2 (9.09%)	11 (50%)	
Grade 3	1 (4.55%)	8 (36.36%)	
After Six Cycles			
No diarrhea	12 (54.55%)	1 (4.55%)	< 0.001 *
Grade 1	6 (27.27%)	3 (13.6%)	
Grade 2	4 (18.18%)	9 (40.91%)	
Grade 3	0 (0%)	5 (22.73%)	
Grade 4	0 (0%)	4 (18.18%)	

Data are presented as frequency (%), *: significant as *p*-value ≤ 0.05.

**Table 4:** Kaplan–Meier survival curve for overall survival.

	Mean	SD	95% CI for the Mean	Mortality	*p*-Value
Glutamine group (*n* = 22)	22.09	3.108	20.51–23.67	5 (22.7%)	0.322
Placebo group (*n* = 22)	20.73	2.986	18.21–23.25	8 (36.4%)	

## Data Availability

Data used and/or analyzed during this study are not available for public access because of patient privacy but are available from the corresponding author upon reasonable request.
